# Causes of Musculoskeletal Pain in Paget’s Disease of Bone

**DOI:** 10.1007/s00223-024-01279-0

**Published:** 2024-09-30

**Authors:** Kathryn Berg, Dervil Dockrell, Lesley Colvin, William D Fraser, Jonathan CY Tang, Terry Aspray, Elaine Dennison, Hrushikesh Divyateja, Nazim Ghouri, Esther Hanison, Richard Keen, Eugene McCloskey, Terence W O’Neill, Faizanur Rahman, Mashood Siddiqi, Stephen Tuck, Jane Turton, Stuart H Ralston

**Affiliations:** 1 Centre for Genomics and Experimental Medicine, Institute of Genetics and Cancer, University of Edinburgh, Western General Hospital, Edinburgh, UK; 2 Division of Population Health and Genomics, Ninewells Hospital and Medical School, University of Dundee, Dundee, UK; 3 Norwich Medical School, University of East Anglia, Norwich, UK; 4 Departments of Clinical Biochemistry, Laboratory Medicine, Diabetes and Endocrinology, Norfolk and Norwich University Hospital NHS Foundation Trust, Norwich Research Park, Norwich, UK; 5 NIHR Newcastle Biomedical Research Centre, Translational Clinical Research Institute, Newcastle University and Newcastle-upon-Tyne Hospitals NHS Trust, Newcastle upon Tyne, UK; 6 MRC Lifecourse Epidemiology Centre, University of Southampton, Southampton, UK; 7 Chemical Pathology, Nottingham University Hospital, Nottingham, UK; 8 Department of Diabetes and Endocrinology, Queen Elizabeth University Hospital, Glasgow, UK; 9 School of Medicine, University of Glasgow, Glasgow, UK; 10 Metabolic Bone Unit, Royal National Orthopaedic Hospital, London, UK; 11 Clinical Medicine, School of Medicine and Population Health, Metabolic Bone Centre, Northern General Hospital, Sheffield, UK; 12 Centre for Epidemiology Versus Arthritis, The University of Manchester and NIHR Manchester Biomedical Research Centre, Manchester University NHS Foundation Trust, Manchester, UK; 13 Department of Chemical Pathology and Metabolic Diseases, University Hospitals of Leicester NHS Trust, Leicester, UK; 14 Liverpool University Hospitals Foundation Trust, Aintree University Hospital, Liverpool, UK; 15 Department of Rheumatology, James Cook University Hospital, Middlesbrough, UK; 16 Department of Geriatric Medicine, University Hospital Llandough, Cardiff, UK

**Keywords:** Paget's Disease of bone, Pain, Osteoarthritis, Macrophage colony stimulating factor

## Abstract

Paget’s disease of bone (PDB) is characterised by increased and disorganised bone remodelling leading to various complications, such as bone deformity, deafness, secondary osteoarthritis, and pathological fracture. Pain is the most common presenting symptom of PDB, but it is unclear to what extent this is due to increased metabolic activity of the disease, complications, or unrelated causes. We conducted a cross-sectional study of 168 people with PDB attending secondary care referral centres in the UK. We documented the presence of musculoskeletal pain and sought to determine its underlying causes. Musculoskeletal pain was reported by 122/168 (72.6%) individuals. The most common cause was osteoarthritis of joints distant from an affected PDB site in 54 (44.3%), followed by metabolically active PDB in 18 (14.7%); bone deformity in 14 (11.4%); osteoarthritis of a joint neighbouring an affected site in 11 (9.0%), neuropathic pain in 10 (8.2%), and various other causes in the remainder. Pain was more common in women (*p*<0.019) and in older individuals (*p*<0.001). Circulating concentrations of macrophage colony-stimulating factor (M-CSF) were significantly higher in those with pain (*p* = 0.008), but there was no difference between groups of patients with and without pain in concentrations of interleukin-6 (IL-6) or biochemical markers of bone turnover. Pain is a common symptom in PDB but is most often due to osteoarthritis at an unaffected site. The study illustrates the importance of fully evaluating people with PDB to determine the underlying cause of pain so that management can be tailored appropriately.

**Supplementary Information:**

The online version contains supplementary material available at 10.1007/s00223-024-01279-0.

## Introduction

Paget’s Disease of the Bone (PDB) is characterised by increased and disorganised bone turnover at one or more skeletal sites. It is now a relatively rare disease which has been estimated to affect up to 0.5% of people over the age of 55 in the UK [1]. Bone pain is the most common reason that people with PDB come to medical attention [2], but the disease is increasingly recognised an incidental finding following blood tests or imaging performed for another reason [2, 3]. Bone pain in people with PDB can arise as the result of increased metabolic activity and in these individuals, the pain often responds to bisphosphonate therapy. Overall, however, there is a poor correlation between metabolic activity of PDB as assessed by measurement of serum concentrations of total alkaline phosphatase (ALP) and the presence of pain in PDB. For example, in the PRISM and PRISM-EZ studies, many individuals with normal circulating levels of ALP continued to experience musculoskeletal pain even after prolonged spells of treatment with bisphosphonates [4, 5]. The reasons for this are unclear but might be due to the fact that musculoskeletal disorders are common in older people, and many people with Paget’s disease may experience pain as the result of an unrelated condition. In order to investigate this issue, we studied the prevalence of pain and evaluated its likely causes in the Pain in Paget’s Disease Study (PiP)—a multi-centre, cross-sectional observational study which recruited participants who were attending eleven secondary referral centres in the UK because of PDB.

## Patients and Methods

### Patients

Participants with a confirmed diagnosis of PDB were recruited from outpatient clinics in the study centres between June 2019 and September 2022. The diagnosis was based on typical radiological features as previously described [6]. Distribution of the disease was determined by radionuclide bone scan.

### Clinical Assessments

Demographic data collected included information on smoking, alcohol intake, age at diagnosis of PDB, family history of PDB, fracture history, analgesic use, bone-targeted treatments, bone deformity, complications related to PDB, and medical comorbidities. The presence or absence of musculoskeletal pain was recorded. This was defined as acute or chronic pain that affected the bones, muscles, ligaments, or tendons. The likely cause of pain was determined by physical examination by one of the co-authors (DD), coupled with a search of the electronic patient medical records to look for a pain diagnosis recorded by the local principal investigator. In order to make a diagnosis of pain secondary to osteoarthritis (OA), there was a requirement to have radiological evidence of OA at the affected site and for the patient to experience joint pain worse on movement. If the cause of pain was not clear following these assessments, the local principal investigator was asked to give their opinion as to the likely cause of pain. This was required in only 6 participants however (3.5%). The response of pain to previous oral or intravenous bisphosphonate treatment was recorded by asking each participant to rate on a five-point scale how well they responded (pain disappeared, a lot better, a little better, no change, worsened). Quality of life was assessed using the Short-Form Survey (SF36) questionnaire [7]. The Leeds Assessment for Neuropathic Symptoms and Signs (LANSS) tool [8] was used to screen for evidence of neuropathic pain. The LANSS tool comprises of a 7-item pain scale. Five of these items are derived from completion of a questionnaire by the patient and two are derived from the results of sensory testing on examination looking for evidence of presence of allodynia and an altered pin-prick threshold. Individuals with a LANSS score of ≥12 are considered to have a neuropathic mechanism contributing to pain.

### Biochemistry

Routine biochemistry was measured by standard techniques at the local hospital laboratories. Creatinine clearance was estimated using the Cockcroft Gault formula. Specialised biochemical markers of bone turnover and cytokines were measured centrally at the Bioanalytical Facility, University of East Anglia. Measurements of Type I collagen C-telopeptides (CTX), Procollagen type I amino-terminal propeptide (PINP), bone-specific alkaline phosphatase (BAP), macrophage Colony-Stimulating factor (M-CSF), and interleukin-6 (IL-6) were measured on serum separated from whole blood. The rationale for measuring these markers and cytokines was to determine if levels of bone turnover and circulating cytokine concentrations were related to the presence of pain. The reason for measuring IL-6 is that it has been previously implicated as a regulatory factor in PDB [9, 10]. The reason for measuring M-CSF is that it is an osteoclastogenic cytokine [11] and might be expected to contribute to pain through this mechanism.

Measurements of CTX were made using an electrochemiluminesence immunoassay (ECLIA) on a Cobas e601 analyser (Roche Diagnostics, Germany). The inter-assay coefficient of variation (CV) for CTX was ≤3% between 0.2 and 1.5 µg/L with a sensitivity of 0.01 µg/L. The reference ranges in men and women combined was 0.16-0.85 μg/L. Measurements of PINP were also made by ECLIA on a Cobas e601 analyser. The PINP inter-assay CV was ≤3% between 20 and 600 µg/L with the sensitivity of 8 µg/L. The reference range in men and women combined was 15.0–76.3 μg/L. Bone-specific alkaline phosphatase (BAP) was measured using the MicroVue enzyme immunoassay (Quidel, Athens, OH, USA). Inter-assay CV for BAP was ≤2.4% up to the concentration of 140 U/L with the lower limit of sensitivity at 0.7 U/L. The reference range in men and women combined for BAP was 11.6-42.7 U/L. Macrophage Colony-Stimulating Factor (M-CSF) and Interleukin 6 (IL-6) were measured by Enzyme-Linked Immunosorbent Assay (ELISA) (Quantikine DMC00B and D6050; Bio-techne R&D Systems, Minneapolis, MN, USA.) according to the manufacturer’s instructions. Inter-assay coefficient of variation (CV) for M-CSF was 3.3–7.4% between the assay lower to upper working limits of 11.7–5000 pg/mL. The manufacturer’s reference range in healthy donors was 180–474 pg/mL. The inter-assay CV for IL-6 was 4.7–8.6% between the assay upper limit of 300 pg/mL and the lower limit of sensitivity at 0.7 pg/mL. The manufacturer’s reference range in healthy donors ranged from 0.7 to 13.9 pg/mL

### Statistical Analysis

Comparisons between subgroups of patients with and without pain were made by Student’s *T* test for continuous variables and chi-square test or Fisher’s exact test for categorical variables. Logistic regression analysis was used to identify independent predictors of pain. The analyses were carried out using SPSS version 29.

### Data Handling

All study data were entered onto a web-based electronic case record form and stored on a REDCap database hosted by the computing team on secure servers at the Institute of Genetics and Cancer.

### Ethics

The study was approved by the West of Scotland Research Ethics Committee 3 (18/WS/0236) and all participants gave written informed consent prior to taking part.

## Results

The clinical and biochemical characteristics of the study population are shown in Table 1. The average age at the time of assessment was 74 years but participants had been first diagnosed with PDB approximately 10 years previously on average. There were a higher proportion of males than females and 8.9% had a family history of PDB. Many individuals had complications of PDB, including bone deformity (30.4%), limb shortening (11.9%), previous pathological fractures (7.1%), and deafness with skull involvement (2.9%). Musculoskeletal pain was present in 122/168 (72.6%) of individuals and osteoarthritis was present in 111/168 (66.1%). In 46 participants, the OA was at a site neighbouring affected bone and in 65 osteoarthritis was at a site distant from Pagetic bone. The most common sites of osteoarthritis were the lumbar spine (26.1%), hands (25.0%), feet (25.0%), hips (20.2%), cervical spine (13.6%), knees (13%), shoulders (12.5%), and thoracic spine (7.7%). One individual (0.6%) had a history of osteosarcoma.

**Table 1:** Clinical characteristics of study population

*Demographics*	
Number of individuals	168
Current Age	74.2 ± 9.8
Age at diagnosis of PDB	64.1 ± 11.2
Male	96 (57.1%)
Family history of PDB	15 (8.9%)
Current smoker	11 (6.5%)
Previous smoker	63 (37.5%)
Alcohol intake (units/week)	6.6 ± 9.9
Body mass index	28.8 ± 5.6
Musculoskeletal pain	122 (72.6%)
*Clinical features*	
Previous bisphosphonate for PDB	92 (54.8%)
Monostotic	107 (63.6%)
Number of PDB-affected bones	1 (1-10)
Bone deformity	51 (30.4%)
Hearing Aid with skull involvement	5 (2.9%)
Limb shortening	20 (11.9%)
Osteosarcoma	1 (0.6%)
Previous fracture through pagetic bone	12 (7.1%)
Spinal stenosis	7 (4.2%)
Osteoarthritis neighbouring a Pagetic site	46 (27.3%)
Osteoarthritis distant from a Pagetic site	65 (38.6%)
*Biochemistry*	
Creatinine clearance	76.4 ± 29.3
Serum 25(OH)D (nmol/L)	68.7 ± 28.9
Serum Total ALP (U/L)	107 ± 66.4
Increased ALP	40/163 (24.5%)
Serum BAP (U/L)	28.9 ± 30.2
Increased BAP	25/164 (15.2%)
Serum CTX μg/L	0.33 ± 0.22
Increased CTX	4/164 (2.4%)
Serum PINP μg/L	72.3 ± 83.6
Increased PINP	40/164 (23.8%)
Serum IL-6 pg/mL	3.2 ± 9.6
Increased IL-6	8/165 (4.8%)
Serum M-CSF pg/mL	428.0 ± 289.3
Increased M-CSF	38/165 (23.0%)

Values are numbers and % or mean ± SD, except for number of affected bones which is median and range. Reference ranges for serum cytokines and biochemical markers of bone turnover are provided in the methods section.

The pattern of skeletal involvement was typical for PDB. In total, 107/168 (63.6%) had monostotic disease and the median number of bones involved was 1, with a range of 1-10. The commonest involved sites were the pelvis (56%), the lumbar spine (20.8%, the femur (20.2%) the skull (12.5%), the tibia (11.3%), the thoracic spine (11.3%), the humerus (5.4%), and the scapula (3.6%). Other sites included the ribs, sternum, clavicle, radius, mandible, maxilla, ulna, patella, and sacrum (13.7%). Just over half of the individuals had previously received bisphosphonates for PDB.

The average estimated creatinine clearance was 76.4 mL/min at the time of enrolment. Serum total ALP was increased above the reference range in 40/163 (24.5%) of individuals, PINP was increased in 40/164 (23.8%), and BAP was increased in 25/164 (15.2%). In contrast CTX was increased in only 4/164 (2.4%). Circulating concentrations of IL-6 were above the reference range in 8/165 (4.8%) of patients. In contrast, the mean circulating concentration of M-CSF was increased above the reference range in 38/165 (23.0%) of individuals. There was no significant difference in circulating concentrations of IL-6 in people who had previously been treated with bisphosphonates and those who had not. Mean ± SD values for IL-6 were 3.36 ± 12.2 pg/ml *vs.* 3.05 ± 5.6 pg/ml, *p*=0.50) and the same was true for M-CSF concentrations (421.1 ± 264.2 pg/ml *vs.* 435.6 ± 315.8 pg/ml, *p*=0.45)

### Causes of Pain

**Fig. 1:**
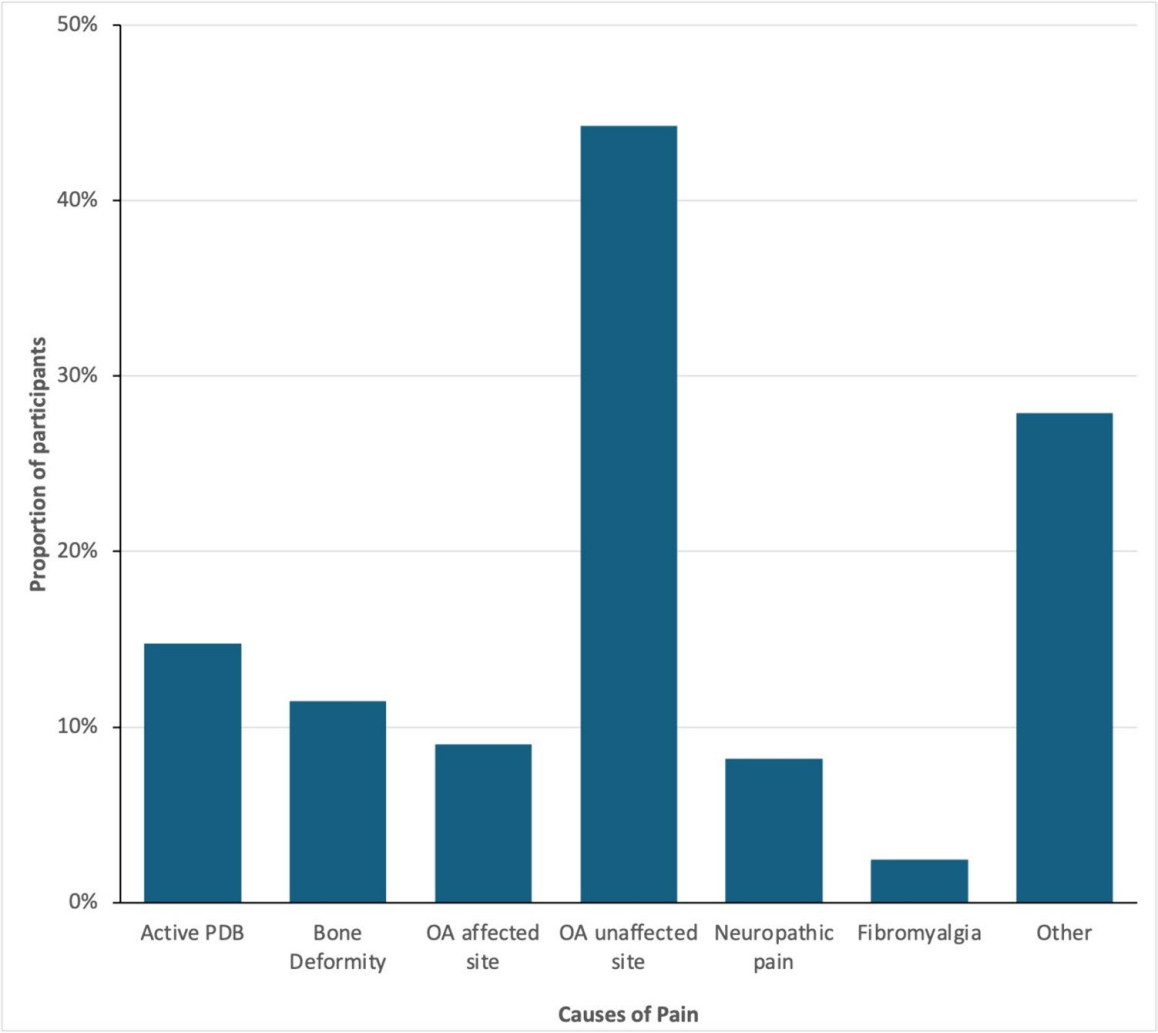
Causes of pain in the study population. The percentages of individuals with different causes of pain are shown. The percentages add up to more than 100% as several individuals had more than one cause of pain. See text for a breakdown of the individual causes of pain in the “other causes” group

The causes of pain in individual participants as assessed clinically are shown in Fig. 1. The most common cause was osteoarthritis at a site distant from affected bone occurring in 54/122 (44.1%), followed by metabolically active PDB in 18/122 (14.7%); bone deformity associated with PDB in 14 (11.4%); and osteoarthritis of joints neighbouring an affected bone in 11 (9.0%). Other causes were neuropathic pain in 10 (8.2%), fibromyalgia in 3 (2.4%), and a wide variety of other causes in the remainder 34 (27.8%), including rotator cuff syndrome, plantar fasciitis, myositis, tendonitis, sciatica or nerve root pain, fractures, recent injuries, and pain following orthopaedic surgery. In 4 individuals, the cause of pain was unknown. In 83/122 individuals, (68.0%) a single cause of pain was identified; in 37 (30.3%) two causes were identified and in 2 individuals, 3 causes were identified.

### Previous Bisphosphonate Therapy

**Fig. 2:**
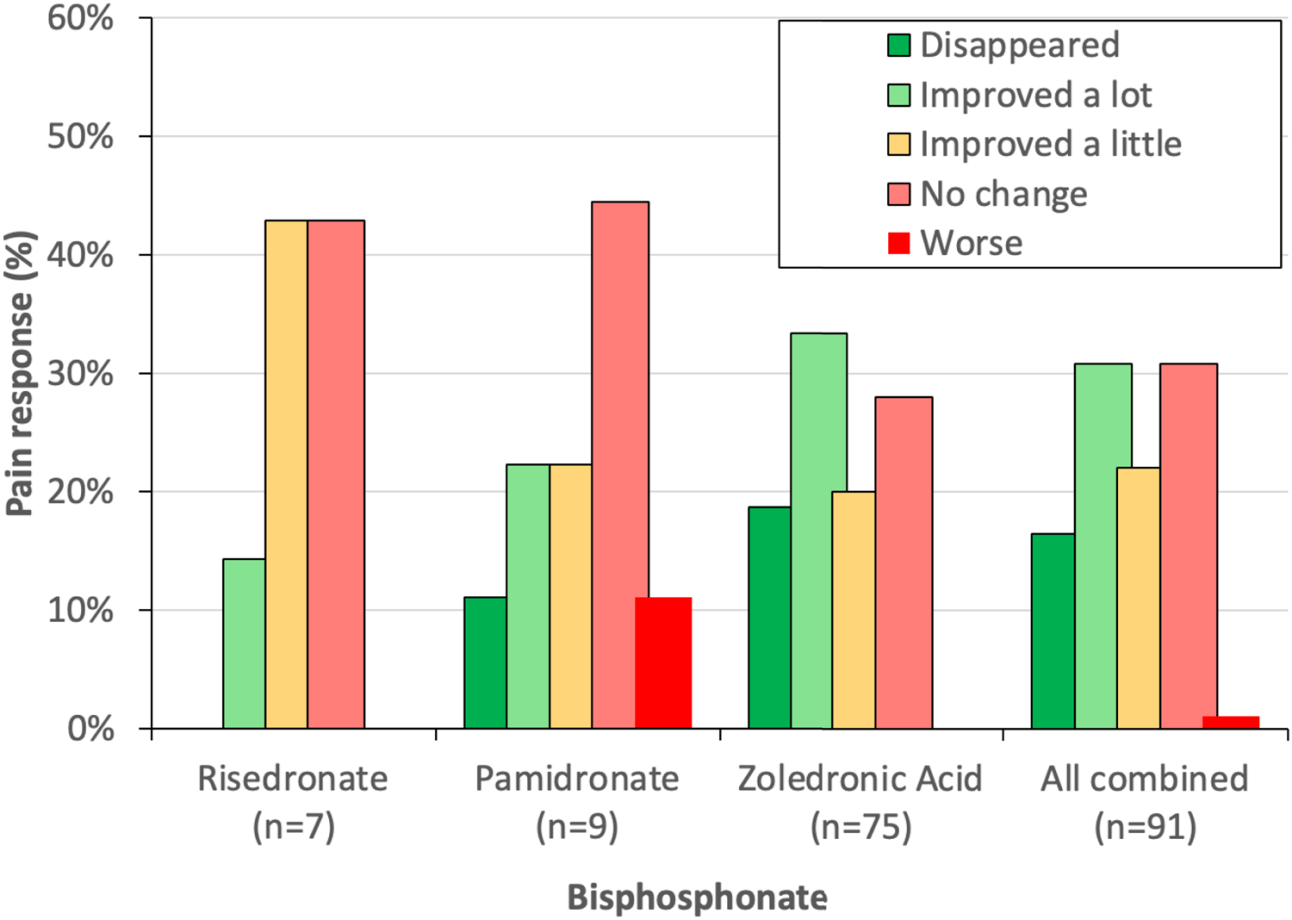
Response of pain to previous bisphosphonate therapy. The values are the proportion of patients who reported that, following bisphosphonate treatment, their pain disappeared, improved a lot, improved a little, did not change, or worsened. The number of individuals in each group and shown on the x-axis

Ninety-two individuals had previously been treated with bisphosphonate therapy but data on response to treatment were available for only 90 of these individuals. The patient-reported response of pain to previous bisphosphonate therapy is summarised in Fig. 2. The most frequently used bisphosphonate was intravenous zoledronic acid 5 mg by infusion (*n*=82) followed by intravenous pamidronate 60 mg by infusion on between one and three occasions (*n*=9) and oral risedronate 30 mg orally for 2 months (*n*=7). Eighty-one individuals had received a single bisphosphonate; 5 had received two different bisphosphonates and 3 had received three bisphosphates. Of the 7 treated with risedronate, 1 (14%) reported that the pain had improved a lot; 3 (43%) that the pain had improved a little; and 3 (43%) reported that it had not changed. For pamidronate, 1 individual (11%) reported the pain had disappeared, 2 (22%) reported it had improved a lot, 2 (22%) reported that it had improved a little, 4 (44%) that it had not changed, and 1 (11%) that it had worsened. For zoledronic acid, 14 individuals (17%) reported the pain had disappeared, 25 (30%) reported it had improved a lot, 15 (18%) reported that it had improved a little, and 21 (26%) that it had not changed. The remaining 7 participants treated with zoledronic acid had not experienced pain before receiving treatment. When data from all bisphosphonates were combined, pain disappeared in 15 (16%), improved a lot in 28 (31%), improved a little in 20 (22%), did not change in 28 (31%), and worsened in 1 (1%). There was no difference in the magnitude of pain response to previous bisphosphonate therapy in the groups of individuals with and without pain overall. However, in an exploratory analysis, we found that 7/16 individuals (44%) who reported that the pain has previously disappeared in response to bisphosphonate therapy were in the no pain group compared with 7/44 (16%) in the current pain group (*p*=0.038, Fisher’s exact test).

### Demographics and Biomarkers in Those With and Without Pain

The demographics, clinical characteristics, and biomarkers in those with and without pain are shown in Table 2. Factors significantly associated with the presence of pain at the level of *p*<0.05 or below were comparing groups of individuals with and without pain were as follows: female sex, increased age, bone deformity, body mass index, the presence of osteoarthritis, and serum M-CSF concentrations. There was no significant association between any of the biochemical markers of bone turnover and the presence and absence of musculoskeletal pain or between IL-6 concentrations and presence or absence of musculoskeletal pain in the study group as a whole. Since many participants had been previously treated with bisphosphonates, we conducted an exploratory subgroup analysis to determine if biochemical markers were associated with pain in participants who had not previously been treated with bisphosphonates. As expected, the circulating concentrations of all markers were higher in this subgroup than in the whole study group, but there was no significant difference in any of the markers in those with and without pain. These results are summarised in supplementary Table 1.

**Table 2:** Demographics, clinical characteristics, and biomarkers in those with and without pain

	No Pain (*n* = 46)	Pain (*n* = 122)	*p* value
Female	13/72 (28.3%)	59/72 (48.4%)	0.019
Age (years)	71.6 ± 10.1	75.2 ± 9.6	0.001
Age at diagnosis	62.6 ± 9.9	64.6 ± 11.6	0.571
Body mass index	27.7 ± 3.8	29.2 ± 6.0	0.022
Bone deformity	0/46 (0.0%)	14/122 (11.4%)	0.012
Limb shortening	2/46 (4.3%)	17/122 (13.9%)	0.080
Osteoarthritis	21/46 (45.7%)	90/122 (73.8%)	<0.001
Metabolically active PDB	2/46 (4.3%)	16/122 (13.1%)	0.101
Serum Total ALP (U/L)	103.7 ± 59.2	109.6 ± 69.2	0.540
Serum BALP (U/L)	25.6 ± 23.2	30.1 ± 32.5	0.394
Serum CTX (μg/L)	0.32 ± 0.21	0.34 ± 0.23	0.672
Serum PINP (μg/L)	68.8 ± 72.5	73.7 ± 87.7	0.722
Serum IL-6 (pg/ml)	3.39 ± 13.9	3.15 ± 7.4	0.482
Serum M-CSF (pg/ml)	346.6 ± 131.4	459.5 ± 325.7	0.008

Values are numbers and % or mean ± SD. The p-values are derived from Students *t* test for continuous variables or Chi-Square test for continuous variables.

Logistic regression analysis was carried out to determine which variables were independent predictors of the presence of pain and of these, only osteoarthritis remained an independent predictor of pain in the study population (*p*=0.019; beta = 0.986, S.E. 0.420, Wald statistic 5.51)

### Quality of Life

When we subdivided participants into groups who had or had not reported pain at the time of study, all subdomains of SF36 were significantly lower in those with pain as compared with those who did not have pain (Table 3).

**Table 3:** Quality of life in those with and without pain

	No Pain (*n* = 46)	Pain (*n* = 122)	*p* value
Physical functioning	79.9 ± 20.7	47.4 ± 31.4	<0.01
Role limitations due to physical health	76.8 ± 34.5	38.5 ± 39.1	<0.01
Role limitations due to emotional health	82.6 ± 35.0	70.8 ± 39.2	0.05
Energy and fatigue	63.7 ± 19.9	47.3 ± 23.8	<0.01
Emotional well-being	82.6 ± 12.6	74.0 ± 19.4	<0.01
Social functioning	86.7 ± 22.9	71.4 ± 29.9	<0.01
Pain	83.5 ± 20.7	48.7 ± 26.1	<0.01
General health	71.0 ± 15.9	55.5 ± 22.3	<0.01

The questionnaire was automatically scored on REDCap and scores were obtained for eight subcategories. A score of 0 indicates the lowest score in that subcategory, whilst a score of 100 would indicate the highest score. The ‘Pain’ group score significantly lower across all of the SF36 subcategories.

## Discussion

The purpose of the PiP study was to evaluate the frequency with which pain occurs in PDB, to determine the likely cause and to identify any biomarkers which are associated with pain. To our knowledge, the PiP study is the only study that has focused on the likely causes of pain in Paget’s disease of bone. In keeping with previous studies [2, 12], pain was a common symptom occurring in 122/168 (72.6%) of participants and those with musculoskeletal pain had reduced quality of life assessed by short-form 36 (SF36) in all domains, as opposed to those who did not have pain.

Our data also show that in most individuals, the pain is not due to increased metabolic activity of PDB but to other causes of which osteoarthritis was the most common. The high frequency of osteoarthritis is not unexpected given that the risk of osteoarthritis is known to be increased in PDB as compared with age-matched controls [13, 14] and the fact that participants were in their seventh decade at the time of assessment. The other disorders that we recorded as causes of pain become increasingly prevalent with ageing and are unlikely to be related to the presence of PDB, but rather to reflect the demographic characteristics of the study population. In this regard chronic musculoskeletal pain becomes increasingly common with age, affecting between 50 and 60% of individuals age 65 years and above [15]. Although the prevalence of pain in our cohort was 72.6%, the present study design does not allow us to determine whether musculoskeletal pain is more common in people with PDB as compared with individuals of a similar age attending secondary care referral centres for conditions other than PDB.

It is important to emphasise that whilst pain related to osteoarthritis occurred most often at sites not directly affected by PDB this does not exclude the possibility that PDB may have had a role on predisposing to osteoarthritis at these sites due to abnormal mechanical loading of joints as the result of deformity or limb shortening or shared predisposing factors for both conditions [14]. Whilst we divided those with osteoarthritis into two groups based on whether neighbouring bone was affected, we acknowledge that having PDB may have been a predisposing factor for both categories of osteoarthritis.

Pain can occur as the result of increased metabolic activity in PDB and this type of pain often responds well to bisphosphonate therapy [16]. Despite this, previous studies have shown that there is a poor correlation between biochemical markers of increased metabolic activity in PDB and the presence of pain. For example, in the PRISM and PRISM-EZ studies [4, 5], concentrations of total alkaline phosphatase were not associated with the presence or absence of musculoskeletal pain and treatment with intensive bisphosphonate therapy in these studies did not improve pain control as compared with symptomatic treatment. Similarly, the randomised comparative trial of zoledronic acid and risedronate in PDB performed by Reid and colleagues [17] showed that whilst both bisphosphonates were very effective at reducing total ALP concentrations, the change in pain scores assessed by the SF36 were much less marked than the ALP response and were below the 5-point threshold that is considered clinically significant. In this study, we observed a poor correlation between biochemical markers of bone turnover and pain overall with no significant difference between the groups of patients with pain or those without pain in circulating concentrations of ALP, BAP, CTX, or PINP. The same was true when we analysed these markers in a subgroup of 81 participants who had not previously been treated with bisphosphonates, although we acknowledge that the number of individuals not previously exposed to bisphosphonates was limited, reducing power to detect possible associations.

Having said that the retrospective data on response of pain to previous bisphosphonate therapy suggested that increased metabolic activity was a contributory factor to pain in many patients, as pain resolved in 16% and improved a lot in 31%. The small improvement in 22% and no change in pain in 31% of participants suggest that in these individuals, other mechanisms of pain most likely predominated. It was also of interest that 7 individuals who reported complete resolution of pain were in the no pain group, indicating that the effects of bisphosphonate therapy on pain in PDB can be long-lasting.

We also measured concentrations of the cytokines IL-6 and M-CSF in the study group as both have previously been implicated as regulatory factors in PDB [9, 18]. This analysis was interesting in two respects. Firstly, we found that IL-6 concentrations were in the reference range in 95% of participants studied, which contrasts markedly with the findings previously reported by Roodman and colleagues who reported serum IL-6 concentrations to be increased approximately tenfold in PDB patients as opposed to controls with an average value of 94.7 pg/ml [9]. The reason for this difference is unclear. In the Roodman paper, it was stated that a bioassay and/or ELISA was used to measure IL-6, although further details were not provided. In order to assess whether the low levels in this series might have been due to previous treatment we compared IL-6 levels in those who had previously been treated by bisphosphonates with those who had not, but no difference was found.

Other investigators have also explored the role of IL-6 in PDB and reported levels similar to those found here with average values of about 3pg/ml with no differences between cases and controls [19, 20]. A study of particular interest is from Rendina and colleagues [10] who looked at IL-6 and other components of it's signalling pathway in relation to pain in a cohort of 85 people with PDB where the disease affected the lumbar spine, pelvis or sacrum [10]. In that study, serum IL-6 concentrations were slightly higher in the PDB group than in the controls but the difference was marginal with an average IL-6 concentration of 3.54 pg/ml in the PDB group, similar to that reported here, compared with 1.81 pg/ml in the control group. Rendina went onto study the relation between circulating components of the IL-6 pathway and pain before and after zoledronic acid treatment. No difference in IL-6 concentrations was observed according to the presence or severity of bone pain before treatment, but concentrations of soluble IL-6 receptor (sIL-6R) were higher in those with bone pain and concentrations of soluble gp130 (sgp130) lower. Additionally, sIL-6rR fell significantly and sgp130 rose significantly 6 months after treatment with zoledronic acid, whereas IL-6 values did not change. Alvarez and colleagues also reported no change in serum IL-6 following tiludronate treatment for PDB patients but did not study sIL-6R or sgp130 [20].

The lack of association between serum IL-6 and pain noted in this study is in accordance with the findings reported by Rendina. However, this does not exclude involvement of the IL-6 signalling pathway as a mediator of pain as previous studies have shown that sIL-6R and sgp130 interact with IL-6 to determine whether IL-6 signalling is activated in target tissues, with the IL-6/sIL-R6 complex acting as an agonist and the IL-6/sgp130 complex acting as an antagonist. This phenomenon is known as IL-6 trans-signalling [21]. The findings of Rendina would be consistent with a model whereby the increased sIL-6R and decreased sgp130 could be responsible for bone pain mediated by activation of the IL-6 receptor even though IL-6 concentrations were unrelated to the presence or severity of pain. We did not measure either sIL-6R or sgp130 in this study but we believe that the role of IL-6 and associated factors as mediators of PDB deserve further study.

It was of interest that serum M-CSF concentrations were increased in about 23% of patients and associated with the presence of pain. It is known that M-CSF plays a key role in osteoclast differentiation and that genetic variations upstream of the *CSF1* gene which encodes M-CSF predispose to Paget’s disease [22, 23]. To our knowledge, this is the first report of elevated M-CSF values in PDB and the first report of an association between levels of this cytokine and pain. Further studies on the role of M-CSF as an autocrine or paracrine mediator of pain in PDB or OA are warranted.

We acknowledge that our study has strengths and weakness. A strength is the fact that this is the only study we are aware of to evaluate the likely causes of pain in PDB. Although previous clinical trials with bisphosphonates have used tools like the short-form 36 (SF36) to look at pain responses these have not attempted to determine whether the pain was thought to be due to PDB or another causes. This is something that would be valuable to look at in future studies. A weakness is that the response to bisphosphonate therapy was evaluated retrospectively and we were unable to assess whether the clinician felt that historic pain was likely to be due to metabolically active PDB or another cause. Despite this, the analysis of response to bisphosphonate treatment supported the results of previous Cochrane reviews [24] and clinical guidelines [16] which have indicated that, of the bisphosphonates in the current use, zoledronic acid is most likely to give a favourable pain response. As the response to bisphosphonates was incomplete we speculate that in many cases this most probably was due to the fact that the patients’ pain was not caused by increased metabolic activity of PDB.

In summary, our study illustrates that when confronted with a patient with PDB who has pain, it is important to consider whether the pain is due to increased metabolic activity of the disease or another cause which may require further investigations and may need to be managed with treatments other than bisphosphonates.

## Supplementary Information

Below is the link to the electronic supplementary material.

Supplementary file1 (DOCX 18 KB)
