# Supplementary material for: Causes of Musculoskeletal Pain in Paget’s Disease of Bone
Source: Calcif Tissue Int. 2024 Sep 30;115(5):533–41. doi: 10.1007/s00223-024-01279-0 (PMC11531417; doi:10.1007/s00223-024-01279-0)
Supplement: Supplementary file 1 — Supplementary file1 (DOCX 18 KB) [file 223_2024_1279_MOESM1_ESM.docx]

**Supplementary Table 1.** **Biochemical markers of bone turnover in those with and without pain not previously treated with bisphosphonates**

|  | No Pain (n = 22) | Pain (n = 59) | p value |
| --- | --- | --- | --- |
| Serum Total ALP (U/L) | 132.9 ± 74.6 | 131.7 ± 87.9 | 0.665 |
| Serum BALP (U/L) | 37.6 ± 29.8 | 40.8 ± 42.2 | 0.363 |
| Serum CTX (μg/L) | 0.45 ± 0.26 | 0.41 ± 0.28 | 0.403 |
| Serum PINP (μg/L) | 105.7 ± 92.4 | 101.1 ± 107.3 | 0.649 |

The values are means and standard deviations. P-values refer to the statistical differences between groups assessed by T-test.
